# Integrated Oral Healthcare Model for Dependent Older Adults: An Experience-Based Co-Design Approach

**DOI:** 10.5334/ijic.9275

**Published:** 2026-05-19

**Authors:** Chanapol Kraitroudpol, Narumanas Korwanich, Kanyarat Korwanich

**Affiliations:** 1 Bureau of Public Health and Environment, Laem Chabang City Municipality, Chonburi, Thailand; 2 Department of Advanced General Dentistry and Dental Public Health, Faculty of Dentistry, Chiang Mai University, Chiang Mai, Thailand

**Keywords:** experience-based co-design, integration of care, oral health, dependent older adults

## Abstract

**Objective:**

To co-develop a stakeholder-informed oral healthcare model for dependent older adults that can be integrated into Thailand’s existing Community-Based Long-Term Care (CBLTC) system using an Experience-Based Co-Design (EBCD) approach.

**Materials and Methods:**

A Participatory Action Research (PAR) approach was employed using Experience-Based Co-Design (EBCD). The process involved five iterative steps: baseline assessment, in-depth interviews, stakeholder-specific focus group discussions, a joint co-design session, and validation of the model. Participants included dependent older adults, formal and informal caregivers, family care teams, local authorities, and private-sector actors (N = 22). Data were collected through qualitative methods and analysed using inductive content analysis to derive the model components and assess stakeholder consensus.

**Results:**

The findings revealed a critical gap in oral healthcare within the current CBLTC framework, despite existing coordination across medical and social care. Participants identified barriers such as a lack of trained personnel, inadequate referral systems, and insufficient caregiver capacity. Through a structured co-design process, a community-specific oral healthcare model was developed. The model defines stakeholder roles, referral pathways, and action plans for implementation.

**Conclusion:**

The co-designed model offers a practical, equity-driven solution to a long-standing gap in Thailand’s long-term care landscape. By embedding oral healthcare into the existing CBLTC infrastructure, the model elevates oral health as a critical component of holistic ageing care. Although further implementation and evaluation are required, this framework sets a precedent for inclusive, community-informed innovation that may inspire broader policy adoption and system-wide integration.

## Introduction

The global population is aging rapidly, and Thailand is among the nations experiencing a significant demographic shift, with a growing proportion of dependent older adults [1]. In response to the challenges posed by an ageing population, the Thai government has implemented a national policy shift from institutional care towards a Community-Based Long-Term Care (CBLTC) model. This model, developed under the Universal Coverage Scheme, is jointly administered by the Ministry of Public Health and the Ministry of Social Development and Human Security [2]. It focuses on enabling older adults to age in place through the integration of health, social, and community resources. The CBLTC model is regarded as one of Southeast Asia’s most advanced examples of decentralised and intersectoral care [3].

However, despite its integrative framework, the system fails to fully incorporate oral health services. Older adults, especially those who are dependent, often experience significant barriers to oral care access. These include difficulty accessing care due to mobility issues, lack of awareness among caregivers, absence of dental personnel in community-based teams, and unclear service pathways [4]. The result is a high burden of untreated oral disease among this population. National surveys and sub-national studies indicate alarmingly high rates of dental caries, periodontal disease, and tooth loss [5,6]. These oral conditions not only diminish quality of life but are also linked to adverse systemic health outcomes. Poor oral hygiene has been identified as a modifiable risk factor for aspiration pneumonia, malnutrition, and poor glycaemic control in diabetic patients, conditions that disproportionately affect older adults [7,8,9]. Therefore, the exclusion of oral health from Thailand’s CBLTC undermines the very principles of integrated care and represents a critical gap in addressing holistic well-being in this vulnerable population.

Integration of oral healthcare into general health systems is now widely recognised as a global priority. The Lancet Commission on Oral Health has called for radical reform, urging that oral health be incorporated into universal health coverage and treated as an essential component of general health to reduce global inequalities [10]. Similarly, the World Health Organization advocates integrating essential oral health interventions into primary care to achieve people-centred, equitable services [11]. This approach is particularly critical for dependent older adults, whose oral health needs are tightly interlinked with chronic disease management, nutrition, and quality of life.

The neglect of oral healthcare within long-term care systems is not unique to Thailand. Globally, countries have struggled to fully embed dental services into ageing care infrastructures. Many interventions designed to improve oral hygiene among older adults in care settings, such as staff education and the involvement of dental professionals have produced mixed results due to methodological weaknesses and inconsistent outcome measures [12]. These international experiences reflect persistent structural and operational challenges in integrating oral health into long-term care frameworks even in high-income contexts.

Thailand’s CBLTC system has been recognised for its collaborative model involving hospital staff, local municipalities, and community health workers. However, oral health remains excluded from its core operations. Within Laem Chabang Municipality—a semi-urban hub with one of the more developed CBLTC networks—routine dental services are notably absent. Although caregivers acknowledge the importance of maintaining oral hygiene, many often lack the necessary skills, training, and time to provide effective support. There are no embedded dental personnel within the care structure, and referral pathways to professional dental care are not clearly established. Oral health is neither screened nor monitored systematically, and care recipients commonly go without preventive or restorative interventions.

This disconnection between systemic health goals and on-the-ground care delivery reveals a critical service gap. Without the deliberate integration of oral health into Thailand’s long-term care frameworks, the system risks falling short in meeting the holistic needs of its most vulnerable older adults. Addressing this gap requires innovative, contextually relevant models that are designed with, and for, the individuals directly involved in caregiving and service provision.

To address the systemic gap in oral healthcare within Thailand’s CBLTC system, this study adopted Experience-Based Co-Design (EBCD)—a participatory methodology grounded in the principles of Participatory Action Research (PAR). EBCD centres on the lived experiences of service users, caregivers, and professionals, positioning them not merely as informants but as active co-creators in the design of services that reflect real-world needs and constraints [13].

EBCD is especially well-suited to contexts where formal systems exist but fail to achieve meaningful integration or user satisfaction. In Thailand, while the CBLTC has successfully coordinated general health and social care through multi-sector collaboration, oral health remains a neglected domain. This marginalisation is due to structural issues such as the absence of dental personnel, fragmented referral pathways, and unclear role delineation among stakeholders. These challenges mirror broader global patterns in ageing societies, where oral care is often marginalised despite its importance for overall well-being [14]. Traditional top-down healthcare planning has not adequately addressed these challenges, partly because it overlooks the daily caregiving realities and implementation bottlenecks at the community level.

By contrast, EBCD enables a bottom-up, dialogical process through which stakeholders collaboratively identify problems, prioritise feasible interventions, and construct context-appropriate service models. It provides a mechanism for bridging evidence-based guidelines with operational realities, fostering a sense of ownership among those directly involved in care delivery [15,16]. While EBCD has shown promise in hospital-based service improvement and aged care in high-income settings, its application to community-based oral health initiatives in lower- and middle-income countries like Thailand remains limited and under-documented. This study therefore seeks to expand its utility into this uncharted, yet critically needed, terrain.

To explore the potential of EBCD in this context, This study aimed to co-develop an operational oral healthcare model for dependent older adults that aligns with Thailand’s existing CBLTC system.

## Methods

This study employed a Participatory Action Research (PAR) design, with a particular focus on the EBCD methodology. PAR was selected for its commitment to collaborative inquiry and cyclical learning, allowing researchers and stakeholders to jointly investigate and act upon real-world challenges. EBCD, nested within PAR, provides a structured framework to capture the lived experiences of stakeholders and translate them into actionable service innovations.

**Figure 1:**
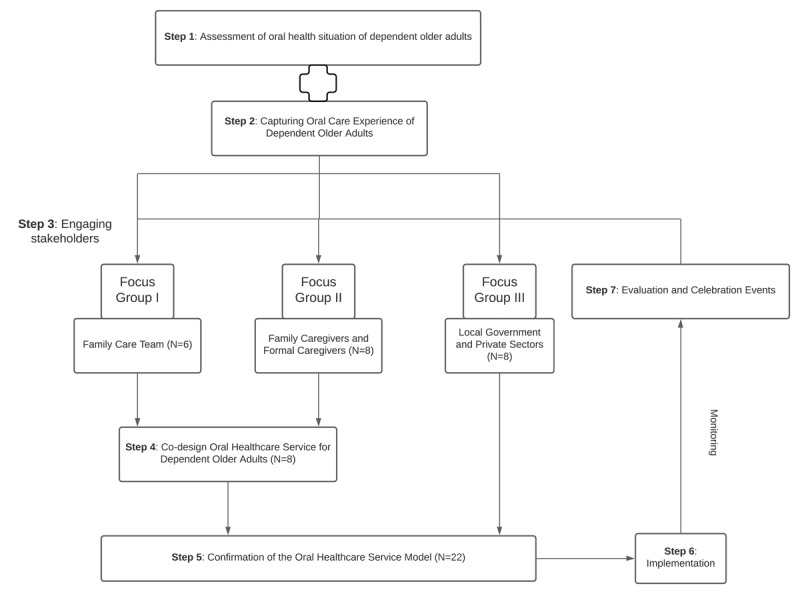
Co-Designing Process Framework.

The EBCD process typically involves six to seven iterative steps, ranging from information gathering through to co-design and evaluation. In this study, we focused specifically on the model development phase, encompassing five sequential steps, as illustrated in the co-designing process framework (Figure 1). These included assessment of oral health status, capturing lived experiences, stakeholder engagement, collaborative co-design, model review and refinement.

The co-designing process was facilitated by the research team and supported by visual and narrative materials from earlier steps (e.g., summary charts, video clips).

### Contextual Background

The study was conducted in Laem Chabang Municipality, Chonburi Province, a fast-growing urban-industrial hub located in eastern Thailand with an estimated population of 200,000 residents. This municipality is recognised as one of the most advanced pilot areas for Thailand’s CBLTC programme, operating under the Universal Coverage Scheme.

As of 2022, Laem Chabang Municipality had an estimated population of approximately 200,000. Of this population, around 8% were older adults aged 60 and above. Among them, 112 individuals were classified as dependent older adults, based on evaluations using the Barthel Index for Activities of Daily Living (ADL) conducted by registered nurses. Only those scoring between 0–11 on the ADL index were formally enrolled in the CBLTC system. It should be noted that not all individuals who meet the criteria for dependency are enrolled in the CBLTC. In some cases, older adults or their families prefer to manage care independently, citing reasons such as privacy, distrust of external services, or a preference for exclusive care by close relatives.

### Existing Care Delivery Model

Thailand’s CBLTC system in Laem Chabang is characterised by a multi-tiered collaborative structure involving medical institutions, municipal agencies, and caregivers at the household level. The system integrates general medical and social care through the coordinated roles of the following stakeholders:

Laem Chabang Hospital: A public-sector facility under the Ministry of Public Health, the hospital provides oversight and clinical support for dependent older adults through the Family Care Team (FCT). This multidisciplinary team is responsible for initial home-based assessments, care planning, and periodic medical visits.

Family Care Team (FCT): Composed of doctors, nurses, and allied health staff, the FCT conducts domiciliary care visits, evaluates functional capacity, and devises personalised care plans for each dependent older adult.

Formal Caregivers: Employed and trained under municipal programmes, formal caregivers are responsible for implementing the FCT’s care plans. Their responsibilities include basic medical care, personal hygiene support, and progress reporting. In Laem Chabang, there were 17 active formal caregivers during the study period.

Informal Caregivers: Typically, family members or relatives, informal caregivers provide day-to-day assistance without financial compensation. They play a vital role in maintaining routine hygiene, feeding, and emotional care, often in coordination with formal caregivers.

In this system, each dependent older adult is assigned a formal caregiver and monitored by the FCT through home visits. The municipality provides caregiver training and logistical support, but oral healthcare is not currently integrated into this care structure. There are no dental professionals embedded in the FCT, and referrals for dental treatment are informal, often requiring patients or families to seek care independently.

### Participants

A total of 22 individuals participated in the co-design process, purposively selected to represent key stakeholder groups involved in the CBLTC of dependent older adults. These were family and formal caregivers (N = 8), FCT members (N = 6), local government and private-sector representatives (N = 8).

Participants were identified through a purposive sampling strategy in collaboration with the local long-term care coordination unit. Selection focused on individuals who either held formal responsibilities or demonstrated leadership within their respective groups, ensuring they could articulate collective experiences and contribute meaningfully to model development.

The family and formal caregiver group included those providing daily in-home care, either as trained employees under the national CBLTC system or as family members offering informal support. The family care team members were healthcare professionals responsible for developing care plans, conducting assessments, and coordinating home visits. Meanwhile, local government and private-sector stakeholders held decision-making roles in programme funding, resource allocation, and policy implementation relevant to the Laem Chabang CBLTC system.

Additionally, three dependent older adults were included during Step 2 of the research (capturing lived experiences), though they did not participate in co-design workshops. They were selected from among the 92 older adults assessed in Step 1 based on their ability to communicate clearly and reflect on their oral health experiences. These narratives were foundational for shaping stakeholder dialogue and ensuring that the voices of older adults remained central to the co-design process.

### Operational definitions

**Dependent older adult:** An individual aged 60 or above with The Barthel Index score for Activities of Daily Living (ADL) between 0–11, indicating a level of functional dependence.

### Data collection

The data collection process followed the structured logic of EBCD, focusing on the five developmental steps adapted for this study. Each step generated qualitative data that informed the subsequent phase of model development. The aim was not simply to gather descriptive insights but to synthesise stakeholder experiences into actionable design features.

#### Step 1 – Assessment of Oral Health Status

The first phase of the study assessed the oral health status, and oral health-related quality of life of dependent older adults residing in Laem Chabang Municipality. This epidemiological investigation laid the foundation for the co-design phase of the integrated oral healthcare service.

A cross-sectional, house-to-house oral examination and structured interview survey was conducted with 92 community-dwelling dependent older adults, registered under the local CBLTC system. The total eligible population was 112 individuals; all were invited, and 92 participated. Inclusion criteria were age ≥60 years and an ADL index score of 0–11. Individuals unable to communicate or uncooperative were excluded.

Oral examinations used standardised clinical tools, including the DMFT (Decayed, Missing, and Filled Teeth) and indicators such as Simplified Oral Hygiene Index (OHI-S). Structured interviews captured data on sociodemographics, health status, oral hygiene behaviour, and self-rated oral health. The instruments were validated by four dental public health experts (IOC = 0.99). Examiner calibration yielded a kappa of 0.83 and 93.75% agreement, confirming reliability.

All data were coded and analysed. Descriptive statistics were used to summarise the findings. To support stakeholder engagement, results were simplified and translated into visual formats and reviewed for accuracy by two independent dental public health specialists.

#### Step 2 – Capturing Lived Experiences

From the 92 dependent older adults who underwent oral health assessments, three were purposively selected for in-depth interviews based on their ability to communicate and reflect effectively. These individuals were not randomly chosen to ensure the narratives would be rich, comprehensible, and useful for the co-design process.

While the sample was small, we ensured diversity of experience by selecting participants with differing levels of dependency and care arrangements. This strategy was designed to capture representative insights from the broader group of 92, while remaining practical within the limits of the EBCD methodology.

These interviews, along with nine others with formal caregivers, family members, and local officials, formed the foundation for a 7-minute edited video summarizing lived experiences, oral health needs, and systemic barriers to care. This video was validated by two subject-matter experts and used as a stimulus in subsequent co-design sessions.

#### Step 3 – Role-Specific Focus Group Discussions

To address potential power imbalances and support open dialogue, three role-specific focus groups were conducted: Family Care Team (N = 6), Family and formal caregivers (N = 8), and Local government and private-sector representatives (N = 8).

Each group was provided with visual and verbal summaries from Steps 1 and 2, including the edited video. Discussions were guided by a semi-structured protocol designed to elicit each group’s perceptions of their roles, challenges, and desired contributions to oral healthcare. Segmenting the groups allowed for candid discussion without hierarchical pressure, especially important given the diverse institutional authority levels involved.

#### Step 4 – Co-Design Workshop

A facilitated co-design workshop was conducted with eight representatives from the earlier caregiver and FCT groups. The session synthesised prior findings and focused on defining stakeholder roles, pre-implementation conditions, performance indicators, and care delivery mechanisms. Participants collaboratively developed draft elements of the oral healthcare model, balancing feasibility, contextual relevance, and equity.

#### Step 5 – Model Confirmation and Validation

The draft model was circulated to all 22 co-design participants for a 7-day validation period. Feedback was gathered via email and follow-up calls. No objections were raised, and minor clarifications were integrated into the final version of the model. While further testing and refinement are still required, the process yielded a stakeholder-endorsed framework suitable for piloting.

### Data analysis

A structured, inductive content analysis approach was used to analyse the qualitative data collected through focus groups, interviews, and observation notes across the five co-design steps. The analysis was conducted in three iterative phases: **(1)** thematic extraction from transcripts and observation notes, **(2)** clustering of themes around emerging concepts such as stakeholder roles, barriers to care, and implementation feasibility, and **(3)** synthesis of findings into components that shaped the structure and content of the proposed oral healthcare model.

A critical objective of this analytical process was to verify whether the emerging model reflected co-designed input across all stakeholder groups. Attention was paid to ensure that insights were equitably incorporated, avoiding the over-representation of perspectives from more dominant voices, such as clinical personnel or policymakers. For instance, caregiver narratives were prioritised in shaping the feasibility of daily oral care tasks, while technical inputs from family care teams informed screening protocols and referral pathways.

This triangulated, feedback-driven validation process helped confirm that the final model was not merely theoretical or top-down but a grounded, collaboratively constructed framework that is responsive to the lived realities and expectations of all stakeholder groups. This analytical rigour strengthens the credibility of the model and reinforces its potential as a scalable prototype for broader implementation within Thailand’s long-term care framework.

### Ethical consideration

Ethical approval for this study was obtained from the Human Experimentation Committee at the Faculty of Dentistry, Chiang Mai University (Reference No. 17/2022). The research adhered strictly to the ethical principles outlined in the Declaration of Helsinki and complied with institutional and national guidelines governing research involving human participants. Data were securely stored and accessed only by the research team. The research team ensured that all procedures minimised risk and maximised respect for participants’ dignity and autonomy throughout the co-design process.

## Results

### Step 1 – Assessment of Oral Health Status

The mean age of participants was 73 years, with ages ranging from 61 to 97 years. The majority were female (70.7%), and the average duration of dependency was 6 years (range: 1–20 years). Nearly all participants were living with at least one non-communicable disease, with hypertension being the most prevalent (72.8%), followed by diabetes mellitus (41.3%). Additionally, 26.1% were post-stroke survivors. Only 5% of participants were not taking any prescription medications, while 22.8% were managing polypharmacy, defined as the use of more than five prescribed medications. All participants were beneficiaries of Thailand’s Universal Health Coverage Scheme.

Clinical findings revealed widespread unmet oral health needs, characterised by high levels of untreated dental decay, and extensive tooth loss. Quantitatively, oral hygiene indices supported these findings: the mean OHI-S score was 3.87 (SD = 1.07), confirming poor oral cleanliness. Participants had an average of 9 remaining teeth and a mean DMFT score of 27.25, underscoring the cumulative burden of disease. Interestingly, despite these clinical indicators of poor oral health, most participants perceived their oral condition as satisfactory (66.3%).

*“My teeth are not good, but I’m used to it. I don’t think I need to see a dentist unless it really hurts.”*

— Dependent older adult, female, age 81.

This quote reflects a common sentiment among participants, in which oral health issues are accepted as normal and not prioritised unless they result in acute pain. This disconnect between clinical need and perceived importance underscores the invisibility of oral health in CBLTC. To support later phases of the study, key data points such as oral hygiene indices were simplified for stakeholders to inform agenda setting and model design.

### Step 2 – Capturing Lived Experiences

In the second step, in-depth interviews were conducted with three dependent older adults and nine key stakeholders across the CBLTC system, including formal caregivers, family members, and local authorities. These interviews were thematically analysed and edited into a 7-minute “trigger film” featuring real-life narratives. The video was produced in Thai, with Thai-language subtitles to ensure accessibility across literacy levels and to maximise familiarity for all stakeholders.

The purpose of the film was to elicit an emotional response and catalyse reflection during subsequent focus group sessions, in accordance with EBCD principles. Participants viewed the film as a shared stimulus before their respective discussions in Step 3.

The trigger film effectively surfaced unspoken concerns and resonated with participants’ lived realities. A family caregiver reflected after viewing the film:

*…“Watching others talk about their struggles made me realise I’m not alone. I’ve been worried I wasn’t doing it right, but now I see we all face similar problems.”*

An informal caregiver added:

*…“It made me feel like my role matters. No one ever asked us how hard it is to clean their mouth every day.”*

The video served as a unifying prompt, levelling power imbalances and prompting deep, personal insights across stakeholder groups. It ensured that the voices of dependent older adults and frontline caregivers were brought to the centre of the co-design process.

### Step 3 – Stakeholder-Specific Focus Groups

Following the narrative interviews and trigger film development, three role-specific focus group discussions were conducted to explore stakeholder perceptions, emotional responses to the video, and their envisioned contributions to oral healthcare. This step aimed to surface differentiated insights based on the functional roles of each group within the CBLTC ecosystem.

#### A. Family Care Team (N = 6)

Consisting of physicians, nurses, and allied health professionals responsible for domiciliary visits and care planning, this group reported a clinical prioritisation of chronic disease management over oral health.

*…“We usually see oral problems only when patients complain of pain. It’s not part of our regular checklist.”*

*…“We need clear guidance and training on how to conduct oral screenings. Right now, it’s not something we are confident doing.”*

They acknowledged systemic gaps in referral pathways to dental care and expressed concern about their limited capacity to intervene in oral health due to a lack of formal protocols.

#### B. Caregivers – Formal and Informal (N = 8)

This mixed group included both trained formal caregivers (N = 4) and informal caregivers (N = 4) providing daily care. They revealed a strong emotional investment in their roles but noted substantial uncertainty regarding oral care techniques and responsibilities.

*…“I want to help her brush her teeth, but I’m afraid she might choke. I’m not sure if I’m doing it right.”*

– Informal caregiver

…*“We get trained in bathing and feeding, but hardly anything about teeth or gums. Maybe we skip it because we’re not sure how.”*

– Formal caregiver

The caregivers called for clearer training and inclusion of oral care in their standard service routines.

#### C. Local Government and Private Sector (N = 8)

This group included municipal health officers and private-sector representatives. They recognised oral health as part of overall well-being but highlighted institutional and regulatory barriers.

…*“There is no government position for a domiciliary dentist in our area, so even if we want to help, we don’t know who should provide the service.”*

– Municipal health officer

*…“We can support supplies and awareness campaigns, but integration needs leadership from health authorities.”*

– Private-sector representatives

Overall, this step confirmed the fragmentation of responsibility and the lack of an oral health mandate across all stakeholder levels. Each group identified oral care as important yet peripheral, within their current roles. These insights were foundational in shaping the collaborative discussions of Step 4.

### Step 4 – Co-Design Workshop

Building on the insights gathered from previous phases, a collaborative co-design workshop was conducted with eight selected participants from the FCT (N = 4) and caregiver group (N = 4). The aim was to translate the emerging themes and shared challenges into a practical and contextually tailored oral healthcare model.

The session opened with a presentation of synthesised findings from Steps 1 to 3, including oral health status, lived experiences, and stakeholder reflections. Participants were encouraged to validate these findings and reflect on how they aligned with their own practices and constraints.

Using guided facilitation, the group jointly explored four domains:

- **Role Identification**: Defining who should be responsible for each component of oral care (e.g., screening, hygiene, referral).
- **Feasibility Assessment**: Evaluating which actions could realistically be integrated into current routines without overburdening stakeholders.
- **Pre-Implementation Conditions**: Identifying enabling factors, such as training, supplies, and interprofessional coordination.
- **Monitoring and Evaluation**: Proposing key performance indicators and feedback mechanisms for model implementation.

Participants reached consensus on several critical elements that would later form the foundation of the proposed oral healthcare model:

- **Screening and Risk Identification**: FCT would be responsible for initial oral health screening during regular domiciliary visits. A simplified checklist and basic training would be required.
- **Daily Oral Hygiene Support**: Formal caregivers would supervise and assist with twice-daily hygiene practices for dependent clients, while informal caregivers would receive training and support to contribute when possible.
- **Domiciliary Dental Referral**: For cases requiring treatment, a pathway was proposed for local government to coordinate with mobile dental units or district dental services.
- **Community Support and Supplies:** Local authorities and private-sector partners would be enlisted to support the provision of oral care supplies and community-level awareness activities.

*…“We need a structure that fits into what we already do—not something new that makes our jobs harder.”*

– FCT participant

*…“If we are trained and supported, we can take care of oral health. But it needs to be simple and practical.”*

– Formal caregiver

The workshop concluded with a jointly developed draft model that included clearly delineated roles, pre-implementation requirements, and monitoring strategies. Participants expressed strong support for the process and its outputs, noting that the co-design session was the first instance in which cross-sector stakeholders had discussed oral health in such an integrated manner.

### Step 5 – Model Refinement and Confirmation

Feedback from this review was minimal and largely supportive. Suggestions focused on minor clarifications in wording and an emphasis on the importance of training and supply availability prior to implementation. These suggestions were incorporated into the final version.

No participant rejected the model or raised substantial objections, indicating a strong level of consensus. Several participants remarked on the collaborative nature of the process:

*…“I feel like this is the first time our voice is part of the system.”*

– Informal caregiver

*…“Now we know our role and how we connect with others—it’s clearer than before*.”

– Local health authority representative

The final model was therefore confirmed with full endorsement from all stakeholder groups. While participants acknowledged that real-world application would require ongoing support and refinement, there was broad agreement that this model provided a meaningful and realistic starting point.

All the steps were executed according to the co-designing process framework (Figure 1). The results of each step are summarised in the table below (Table 1).

**Table 1:** Summary of Results for Each Step of the Co-Designing Process.


STEPS	METHODS	PARTICIPANTS	RESULTS/CONCLUSIONS

1. Assessment of oral health situation	Oral examination and interviewing	Dependent older adults (N = 92)	Poor oral health status but minimally impacted their quality of life

2. Capturing oral care experience	Deep interview with video recording	Dependent older adults (N = 3) and stakeholders (N = 9)	Edited film highlighting real-life oral care experiences of stakeholders, used as a discussion resource.

3. Engaging stakeholders	3 Independent focus group discussion (mainly discussed about each experience and each subgroup ideas of their contribution to oral health of dependent older adults)	3.1 Family care team (N = 6)	The oral care was not prioritised due to general care workload and lack of a referral dentist. Desired roles included performing oral health screenings.

		3.2 Caregivers (N = 8)	Recognised the importance of oral health but were unsure about their skills. They wanted to ensure they could perform oral hygiene care correctly and safely, without causing harm.

		3.3 Local government and private sector (N = 8)	Strong perception of the importance of oral health in CBLTC. The exclusion of oral health was due to the absence of a government-operated dentist. They were willing to provide financial and bureaucratic support for integrating oral healthcare.

4. Co-design oral healthcare service	Focus group discussion	Representatives from previous FCT and caregiver subgroups (N = 8)	The goal was to establish comprehensive oral healthcare for dependent older adults, ensuring emergency dental needs were met, along with regular screenings and quality hygiene care, with caregiver support or independently, as outlined in the final co-designed model (Figure 2).

5. Confirmation of the oral healthcare model	Reviewing	All co-designed members (N = 22)	The final model was shared with all members for a 7-day confirmation period. No rejections were received, indicating unanimous approval.


**Figure 2:**
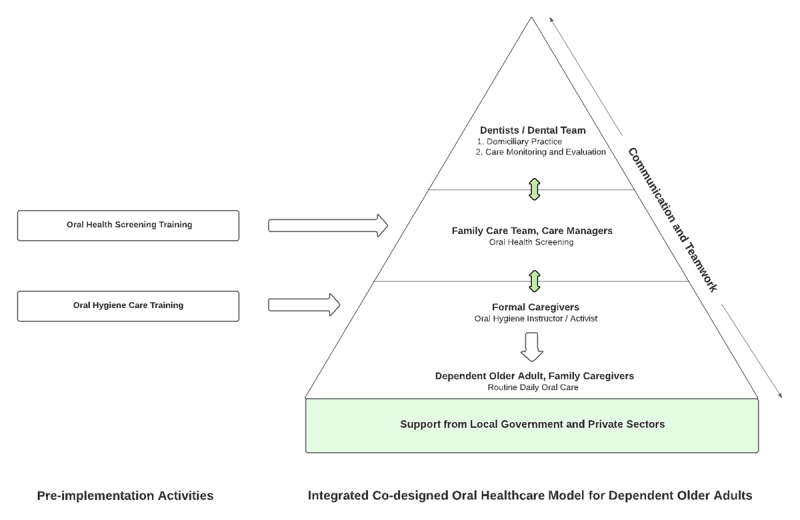
Final Co-Designed Oral Healthcare Model.

Upon completing the process, the final co-designed oral healthcare model was established (Figure 2), comprising two distinct components: Pre-Implementation Activities and the Oral Healthcare Model. The Pre-Implementation Activities outlined essential prerequisites preceding the actionable steps, presented in the form of a pyramid-shaped hierarchy. This structure encompassed all stakeholders, delineating their respective roles and responsibilities.

At the foundation of the pyramid, robust support from the local government and corporations served as the initiative’s cornerstone. Their commitment and resources were pivotal to the programme’s success. Ascending the pyramid, formal and informal caregivers appeared next, pivotal in daily oral hygiene care for dependent older adults. Above them were the family care team members, facilitating oral health screening.

Continuing upward, the pyramid’s pinnacle represented the dental team, led by dentists. Here, clinical expertise and academic knowledge converge, providing essential dental care.

The pyramidal model highlighted the essential collaboration among stakeholders, emphasizing their shared commitment to delivering integrated oral healthcare services. Importantly, the shape did not imply a hierarchy of authority but symbolised the convergence of diverse efforts. The programme’s success depended on the collective dedication of all participants.

The model aimed to ensure that all dependent older adults would undergo screenings by the family care team, with emergency cases or those requiring treatment receiving domiciliary dental care provided by a dentist. Daily oral care was assigned to informal caregivers, who would be trained in oral health practices by formal caregivers equipped with specialised training.

Crucially, the pyramidal structure represented unity rather than hierarchy, underscoring the unique contributions of each stakeholder. The stakeholders unanimously endorsed the integrated, co-designed oral healthcare model, affirming its accuracy and readiness for real-world application. Moving forward, the next phase involves implementation the plan to enhance oral healthcare for dependent older adults. This collective endorsement marked a significant step toward improved oral health outcomes for the community.

The EBCD process fostered meaningful collaboration by deliberately addressing power asymmetries between participant groups. By segmenting stakeholders during Step 3 into separate focus groups: family care teams, caregivers, and local authorities, the process enabled participants to contribute without hierarchical pressure. Each group articulated concerns and proposed roles aligned with their real-world capabilities and limitations.

The co-design step (Step 4) further facilitated this inclusivity by synthesising feedback across the three groups, leading to a unified service framework that all participants agreed upon. Informal caregivers felt validated in their contributions, formal caregivers gained clarity regarding their scope of responsibilities, and policymakers recognised gaps in oral care that had previously gone unacknowledged.

Moreover, the model reflects a shift from passive service delivery to proactive engagement. Rather than relying solely on external dental professionals, the model promotes decentralised care anchored in community actors, making integration feasible within existing long-term care pathways. The outcome is not a static service blueprint but a flexible, community-anchored system that can adapt to local conditions while maintaining fidelity to core oral health standards.

As one caregiver shared in the final validation workshop:

…*“Before, we didn’t think oral care was something we had to worry about. But when we talked about our role, we realised we are the ones with them every day. If we don’t do it, no one will.”*

This reflection underscores the transformative impact of participatory design—not only in shaping services, but also in reshaping stakeholder awareness, ownership, and long-term sustainability.

## Discussion

This study provides the first participatory and comprehensive approach to developing an integrated oral healthcare model for dependent older adults within Thailand’s CBLTC system. The integration of oral health into CBLTC has been largely underexplored in Thailand and internationally. This research is notable for employing EBCD, involving stakeholders from multiple levels, including dependent older adults, caregivers, and healthcare professionals. This methodological choice enabled the development of a highly contextual, inclusive, and relevant model of oral healthcare for this vulnerable population. The originality of this study lies in its ability to balance evidence-based guidelines with the lived experiences of those directly involved in care, resulting in a practical and actionable framework for integrating oral health into CBLTC systems.

The primary strength of this research lies in its emphasis on participatory co-design, which fosters collaboration among stakeholders at all levels. EBCD allowed for the involvement of diverse stakeholders, from family caregivers to local government representatives. The need for such participatory models in healthcare systems, especially in the long-term care sector, is well established [17]. As healthcare systems evolve, especially in low- and middle-income countries, the inclusion of end-users, such as caregivers and dependent older adults is crucial for developing solutions that are both feasible and sustainable [18]. This study highlights the value of co-design in addressing systemic gaps that are often overlooked in traditional healthcare approaches. By focusing on community engagement and stakeholder ownership, the study creates a model that is deeply rooted in the local context and responsive to the challenges faced by caregivers and healthcare providers.

The use of EBCD in this study was not without challenges. A key adaptation involved differentiating focus group discussions into three distinct stakeholder subgroups: family care teams, informal caregivers, and local government/private sector representatives. In standard EBCD processes, participants from various backgrounds often engage in mixed-group discussions. However, in our study, power dynamics within the groups were a significant concern. Healthcare professionals, who often possess greater technical expertise, risked dominating the conversation, leaving informal caregivers, who are typically less formally trained, feeling marginalised. Therefore, the co-design process involved subgroup discussions to allow each group to express their views freely without hierarchical pressures [19]. This was crucial for eliciting the unique perspectives of informal caregivers, who are often the primary providers of care but may feel less confident in healthcare discussions [20]. The results of these discussions were then integrated to form a balanced, holistic model that took into account all perspectives.

In alignment with concerns regarding power asymmetries, the research team, comprising dentists, intentionally adopted a facilitative rather than directive role during the co-design process. While dentists were consulted during the preparatory stage, they were purposefully excluded from the core co-design activities. This decision was both methodological and philosophical: it sought to avoid reproducing the top-down dynamics that have characterised previous oral health initiatives. By consciously stepping back, the researchers sought to avoid overshadowing participants’ contributions and to foster a space in which caregivers and community actors could articulate priorities grounded in their own lived realities. The resulting model was thus shaped not by professional assumptions, but by the practical wisdom of those most intimately involved in providing daily care. This participatory stance was essential to promoting ownership, contextual relevance, and long-term feasibility, particularly in light of past guidelines which despite clinical rigour, frequently failed to translate into practice due to insufficient stakeholder involvement during their formulation.

When comparing this model with existing healthcare frameworks, it becomes clear that the participatory approach offers several advantages over traditional top-down models. Existing strategies and models, such as those reviewed by Weening-Verbree et al., rely on standardised protocols and clinical research to guide implementation. While these approaches have proven effective in some contexts, they often fail to account for the unique needs and lived experiences of the populations they serve [21].

In contrast, This co-designed oral healthcare model presents a significant departure from conventional approaches by embedding oral health within Thailand’s CBLTC system. Unlike existing models, this model was co-created with all stakeholders. It prioritises daily care routines, prevention, and community capacity over specialist treatment. This participatory and flexible design not only enhances contextual relevance and ownership but also addresses long-standing barriers to guideline adoption, such as lack of caregiver involvement and impracticality in real-world settings. Table 2 below summarises the key differences.

**Table 2:** Comparative Features of Conventional Oral Healthcare Models/Protocols and the Co-Designed Oral Healthcare Model.


	CONVENTIONAL MODELS/PROTOCOLS	CO-DESIGNED MODEL

**Leadership**	Dentist-led, expert-driven	Community-led

**Care Setting**	Clinic-based or facility-based	Home-based, integrated into CBLTC system

**Approach to Intervention**	Treatment-focused, episodic	Daily care, prevention, early detection

**Stakeholder Involvement**	Limited	Active involvement of all stakeholders

**Guidelines & Tools**	Formal, technical	Simple, visual, co-created tools

**Training Delivery**	Specialist-led, lecture-based	Peer-led, context-specific

**Adaptability**	One-size-fits-all protocols	Flexible, responsive to local context and feedback

**Sustainability**	Resource-intensive, dependent on professionals	Utilises existing community care structures


It is important to emphasise that this model does not seek to replace or diminish the value of conventional clinical protocols, which remain essential in many care settings. Rather, it offers an alternative lens through which oral healthcare can be reimagined, particularly for populations where access to specialist-driven care is limited. By aligning with the principles of community engagement and contextual adaptability, this co-designed model complements existing frameworks and bridges longstanding implementation gaps. In doing so, it adds a new layer of practicality and ownership that enhances—rather than competes with—formal guideline-based approaches.

What sets our co-designed oral healthcare model apart is its community-based approach. A distinguishing feature is its flexibility, as it incorporates feedback from stakeholders in real time and adapts to the community’s specific needs. This approach not only fosters a sense of ownership among stakeholders but also ensures that the model remains responsive to local contexts, making successful implementation more likely. Moreover, the model integrates oral healthcare into existing care pathways, highlighting the importance of multi-sector collaboration in addressing the full range of health needs among older adults.

Despite its innovative approach, this study is not without limitations. One major challenge is ensuring the long-term sustainability of the co-designed model. Given the reliance on trained healthcare personnel, particularly dentists, for domiciliary care, the availability of these resources in rural or resource-constrained areas may pose a barrier.

Additionally, maintaining stakeholder engagement over time is crucial for the success of the model, but may prove difficult without ongoing funding or institutional support. Another limitation is the study’s geographic and demographic focus, conducted in Laem Chabang Municipality, Chonburi Province, which may limit its generalizability to other regions of Thailand or to countries with different healthcare systems.

A key methodological limitation lies in the qualitative nature of the data collected. While qualitative methods, such as interviews and focus groups, provide rich insights into stakeholders’ perspectives, they are also subject to interpretation bias. The small sample size, while ensuring in-depth exploration of stakeholder views, limits the generalizability of the findings. Additionally, the time and resource-intensive nature of the co-design process could hinder the feasibility of replicating this approach in low-resource settings.

The implications of this study for practice and policy are significant. The findings underscore the need to integrate oral healthcare into long-term care systems, particularly in community-based settings. Policymakers and healthcare providers should consider adopting participatory approaches, such as EBCD, to design and implement integrated healthcare models that are context-sensitive and sustainable. By incorporating oral health into the broader framework of long-term care, these models can enhance the quality of life for older adults, reduce the incidence of oral health-related systemic conditions, and ultimately lead to more holistic, person-centred care. This study demonstrates that integrating oral health into long-term care can be achieved through collaboration, stakeholder engagement, and context-specific adaptations, providing a roadmap for future healthcare innovations.

Moving forward, the model developed in this study requires real-world testing and further refinement to assess its effectiveness. Implementation trials, followed by longitudinal studies, will be essential for evaluating the impact of the model on oral health outcomes and the overall quality of life of dependent older adults. Furthermore, adapting the model for use in different cultural and healthcare contexts, both within Thailand and globally, will be crucial for its broader applicability. Policymakers, healthcare providers, and researchers should continue to explore ways to integrate oral health into long-term care systems, particularly in resource-limited settings, to ensure that all older adults receive comprehensive care.

While this study focused on dependent older adults, the participatory principles and methods applied, particularly EBCD, are highly adaptable to other populations and healthcare settings. EBCD and similar stakeholder-driven approaches have been effectively used in areas such as mental health care, maternal health, and chronic disease management [22]. This highlights their potential for addressing service gaps in a wide range of contexts where clinical care may not align well with users lived experiences.

Extending this potential, the co-design approach may offer meaningful benefits for other underserved groups, such as individuals living with disabilities. By centring the voices of those directly affected, community-informed models like the one proposed here provide a practical route toward more inclusive and person-centred healthcare.

## Conclusion

Rooted in local wisdom and developed through inclusive collaboration, this study presents a practical oral healthcare model for dependent older adults within Thailand’s CBLTC system. By applying the EBCD framework, the study amplifies the voices of groups often excluded from healthcare planning, including caregivers, dependent older adults, and community-level practitioners. The result is a care model that aligns with both lived experiences and structural service gaps. Although the model has not yet been implemented, it provides a strong foundation for improving equity in geriatric care by positioning oral health as an essential and integrated element within the long-term care continuum.
